# Biochemical and immunological characterisation of Act d 10 a lipid transfer proteins from green kiwifruit

**DOI:** 10.1186/2045-7022-1-S1-O23

**Published:** 2011-08-12

**Authors:** Maria Livia Bernardi, Ivana Giangrieco, Laura Camardella, Maria Rosaria Panico, Danila Zennaro, Rosetta Ferrara, Paola Palazzo, Lisa Tuppo, Maurizio Tamburrini, Vito Carratore, Mario Santoro, Maria Antonietta Ciardiello, Adriano Mari

**Affiliations:** 1 IDI-IRCCS, Center for Molecular Allergology, Rome, Italy; 2 C.N.R., Institute of Protein Biochemistry, Naples, Italy

## Aims

The aim of this study was to assess the presence of a Lipid Transfer Proteins (LTP) in green kiwifruit (Actinidia deliciosa) and to evaluated the allergenic activity of the newly identified protein.

## Methods

Pulp and seeds of green kiwifruit were manually separated and homogenized. Act d 10 was purified from seed extracts by ion exchange chromatography and RP-HPLC, and sequenced by an automatic amino acid sequencer. Act d 10 was evaluated in vivo by skin prick test (SPT) and in vitro by detecting IgE using streptavidin-conjugated CAP (Phadia, Sweden). Act d 10 was spotted on a customized ISAC microarray (PMD, Austria) for evaluating IgE prevalence in a larger cohort of allergic patients.

## Results

The purification procedure allowed to obtain about 0.4 mg of natural Act d 10 from 1 g of kiwifruit seeds. The primary structure comprises 92 amino acids with a molecular mass of 9,458 Da. Homology by BLAST search in Uniprot database showed Act d 10 displaying the higher identity (56%) with an isoform of Ara h 9. Act d 10 shares 47% of overall sequence identity with Pru p 3. However, higher levels of residue conservation are detected in some regions. Seven patients selected with clinical history of kiwifruit allergy and specific IgE to Pru p 3 were positive to Act d 10 by SPT; the same patients had specific IgE to Act d 10 as detected by the CAP system. 804 randomly selected allergic patients were tested for IgE to Act d 10 and 170 of them (21.14%) were detected positive; 152 of these 170 patients (89.41%) had IgE to Pru p 3.

## Conclusions

Act d 10 is a new LTP isolated from Actinidia deliciosa, contained in significant amounts in the green kiwi seed. The sequence identity between Act d 10 and other already known allergenic LTPs is not very high, however the significant conservation of some epitope regions suggested potential IgE cross-reactivities.The IgE binding activity of sera from clinically allergic patients has been shown by SPT and direct IgE detection on two different systems. IgE co-recognition of kiwi LTPs and other known LTPs seems to be dependent on the epitope distribution on the different allergens.

